# Data Resource Profile: The National Dental Centre Singapore Oral Disease Registry (NDCS-ODR)

**DOI:** 10.1093/ije/dyag171

**Published:** 2026-08-18

**Authors:** Marco Aurélio Peres, Pinyan Liu, John Rong Hao Tay

**Affiliations:** Programme in Health Services Research and Population Health Research Programme, Duke-NUS Medical School, Singapore, Singapore; National Dental Research Institute Singapore, National Dental Centre Singapore, Singapore, Singapore; Centre for Biomedical Data Science, Duke-NUS Medical School, Singapore, Singapore; Programme in Health Services Research and Population Health Research Programme, Duke-NUS Medical School, Singapore, Singapore; National Dental Research Institute Singapore, National Dental Centre Singapore, Singapore, Singapore

**Keywords:** longitudinal studies, electronic health records, missing data

Key Features

- The National Dental Centre Singapore Oral Disease Registry (NDCS-ODR) was established to create a standardized, longitudinal repository of routinely collected clinical oral health data, with the dual aim of supporting epidemiological and health services research and serving as a methodological platform for addressing hierarchical, outcome-dependent missingness in real-world electronic health records.
- The registry is based at the National Dental Centre Singapore, a national tertiary referral centre, and includes all patients attending between January 2013 and June 2025; as of the most recent extraction, it comprises 229 249 patients with a mean age of 49.1 years, 50.1% male, and a composition reflecting Singapore’s multi-ethnic population.
- Follow-up is driven by routine clinical care rather than a fixed protocol, yielding irregular visit intervals with a median of two visits per patient.
- The registry sits within the SingHealth data ecosystem, enabling linkage with other clinical datasets for oral–systemic and multimorbidity research.
- Data captured include demographics, the Singapore Housing Index as a socioeconomic proxy, smoking status, diagnoses, odontograms, and high-resolution periodontal charting (probing pocket depth [PPD], gingival recession [REC], clinical attachment level [CAL], bleeding on probing [BOP]) at up to 192 sites per visit.
- Access requires proposal submission to the ODR Executive Committee (ndris@ndcs.com.sg).

## Data resource basics

### Background and rationale

The rapid growth of electronic health records (EHRs) has changed how we do epidemiological research. These records enable the study of disease patterns, trends, and health system outcomes in far greater detail than before. However, the ways we can analyse this data are often limited by two linked problems that arise in regular medical care: complicated data organization and many missing entries. These issues make describing and interpreting results more difficult, especially when data are collected at different biological levels and when what is recorded depends on clinical choices [1, 2].

Oral health provides a particularly illustrative example. Conditions such as periodontitis are assessed through detailed clinical examinations that generate high-dimensional data, with measurements recorded at multiple anatomical sites within each tooth and repeated across time. In practice, complete data capture is rarely achieved. Clinical workflows, time constraints, patient tolerance, and diagnostic priorities all influence which measurements are recorded. As a result, systematic, often non-random patterns of missingness emerge. This form of structured incompleteness, inherent to real-world clinical data, poses important challenges for valid inference. It requires methodological approaches that explicitly account for the data-generating process. In Singapore, oral diseases remain highly prevalent and represent an important component of the non-communicable disease (NCD) burden, particularly in the context of rapid population ageing [3–9]. The National Dental Centre Singapore Oral Disease Registry (NDCS-ODR) was established within the SingHealth data infrastructure to create a standardized and longitudinal repository of oral health data derived from routine clinical care, with the dual purpose of supporting questions, and enabling methodological advances in the analysis of real-world data. SingHealth is Singapore’s largest public healthcare cluster, comprising acute hospitals, national specialty centres, community hospitals, and polyclinics. Dental services captured within the SingHealth ecosystem include specialist outpatient dental care provided by the National Dental Centre Singapore (NDCS) and SingHealth polyclinics. However, dental care nationally is also provided by private dental practices, private specialist clinics, and other public institutions outside the SingHealth cluster (National Healthcare Group and National University Health System). The NDCS-ODR therefore represents patients receiving care within the NDCS rather than the entirety of dental care delivered in Singapore.

A central motivation for the development of the NDCS-ODR is the recognition that EHR-based data are incomplete, and often in systematic and informative ways. In everyday care, what gets measured often depends on how sick someone is, what clinicians think, and how people use health services. These patterns in missing data can skew our estimates of how often conditions occur or how they change over time if not handled carefully [10, 11]. By clearly describing and tracking these patterns and using advanced tools, such as grouped methods for filling in missing data over time, the registry aims to improve answers to questions in oral health research and to help develop better ways to study real-world data.

### Setting, population, and follow-up

The NDCS is one of two national tertiary referral centres for specialist dental care in Singapore and receives referrals from both public and private dental providers for specialist management. NDCS-ODR includes both children and adults receiving care at NDCS. As the registry is based on patients attending a tertiary specialist centre, it is not population-based and does not capture all dental care provided in Singapore. Routine dental care is also provided by private dental clinics and by institutions within the other two public healthcare clusters.

The NDCS-ODR includes all individuals attending the centre between January 2013 and June 2025. As of the most recent data extraction, the registry comprises 229 249 unique patients, with a mean age of approximately 49 years and a near-equal distribution of males and females (Table 1). The cohort consists of nearly equal numbers of males and females. Demographically, 77.6% of patients are Chinese, 8.7% Malay, 8.4% Indian, and 5.4% from other ethnicities, with 93% being Singapore citizens. Other residency groups are also represented.

**Table 1:** Baseline characteristics of the NDCS-ODR cohort (*N* = 229 000).

Characteristic	*N* or value^a^	%
Demographics		
Total patients	229 249	
Age at first visit, mean (SD)	49.1 (19.5)	
Sex		
Male	114 943	50.1
Female	114 306	49.9
Ethnicity		
Chinese	177 827	77.6
Malay	19 842	8.7
Indian	19 362	8.4
Other	12 218	5.3
Residency status		
Singapore citizen	212 000	92.5
Permanent resident	11 253	4.9
Other	5991	2.6
Missing	5	0.0
Socioeconomic and behavioural		
SHI		
<3.0	14 785	6.4
3.0 to <4.0	38 372	16.7
4.0 to <6.0	91 347	39.8
6.0	82 900	36.2
Missing	1845	0.8
<3.0	14 785	6.4
Smoking status (at first visit)		
Current	16 697	7.3
Never	187 780	81.9
Former	5780	2.5
Not recorded	18 992	8.3

a Values are presented as *N* (%) for categorical variables and mean (SD) or continuous variables. Baseline is defined as the first recorded visit for each patient. SHI is a building-level socioeconomic indicator based on the weighted average number of rooms per dwelling within a residential building (range 1–6), with higher values indicating higher socioeconomic position.

SHI: Singapore Housing Index; SD: standard deviation.

Patients present with a wide spectrum of oral health conditions, consistent with the centre’s referral role. Accordingly, the cohort is not intended to be representative of the general population but of patients receiving specialist care, a distinction with other clinical registries resources [12–14].

Follow-up is determined by clinical need rather than by a predefined study protocol, reflecting real-world patterns of care. Among patients with periodontal data, individuals contribute a median of approximately two visits, with some patients contributing substantially longer (Fig. 2). This design captures dynamic processes in oral health but introduces irregular and outcome-dependent observations (e.g. patients with more severe disease, higher risk profiles, or ongoing treatment needs are more likely to be seen more frequently), requiring methodological approaches that explicitly address this [15, 16].

### Funding and follow-up plans

Researchers analysing NDCS-ODR data pay a fee that covers the maintenance of the registry and virtual research environments. Data are handled in accordance with the Singapore Personal Data Protection Act, the Human Biomedical Research Act, and the Declaration of Helsinki. The registry is intended to collect routine data indefinitely.

## Data collected

### Data process from source to registry

The NDCS-ODR is derived from the structured electronic dental record system used across NDCS. Data are extracted periodically, de-identified, and stored within secure SingHealth computing environments. Patient identifiers are replaced with pseudonyms through institutional trusted-third-party procedures, and access is restricted to authorized users within the secure analytical environment.

### Data quality measures

Quality assurance is performed at both extraction and analysis stages. At extraction, structural checks verify table completeness, variable formats, and consistency of coding schemes across time, with particular attention to transitions between the 1999 Classification of Periodontal Diseases and Conditions and 2018 European Federation of Periodontology/American Academy of Periodontology periodontal classification systems. Quantitative checks compare patient counts, visit counts, and variable distributions against prior extractions, flagging anomalies for manual review. Before analysis-ready datasets are produced, additional checks examine within-patient consistency, distributional outliers in continuous periodontal measurements, and duplicate records. Data are otherwise left minimally processed, with decisions on handling missingness and measurement error delegated to individual research teams in consultation with the ODR methodological support team.

### Data types and structure

**Figure 1:**
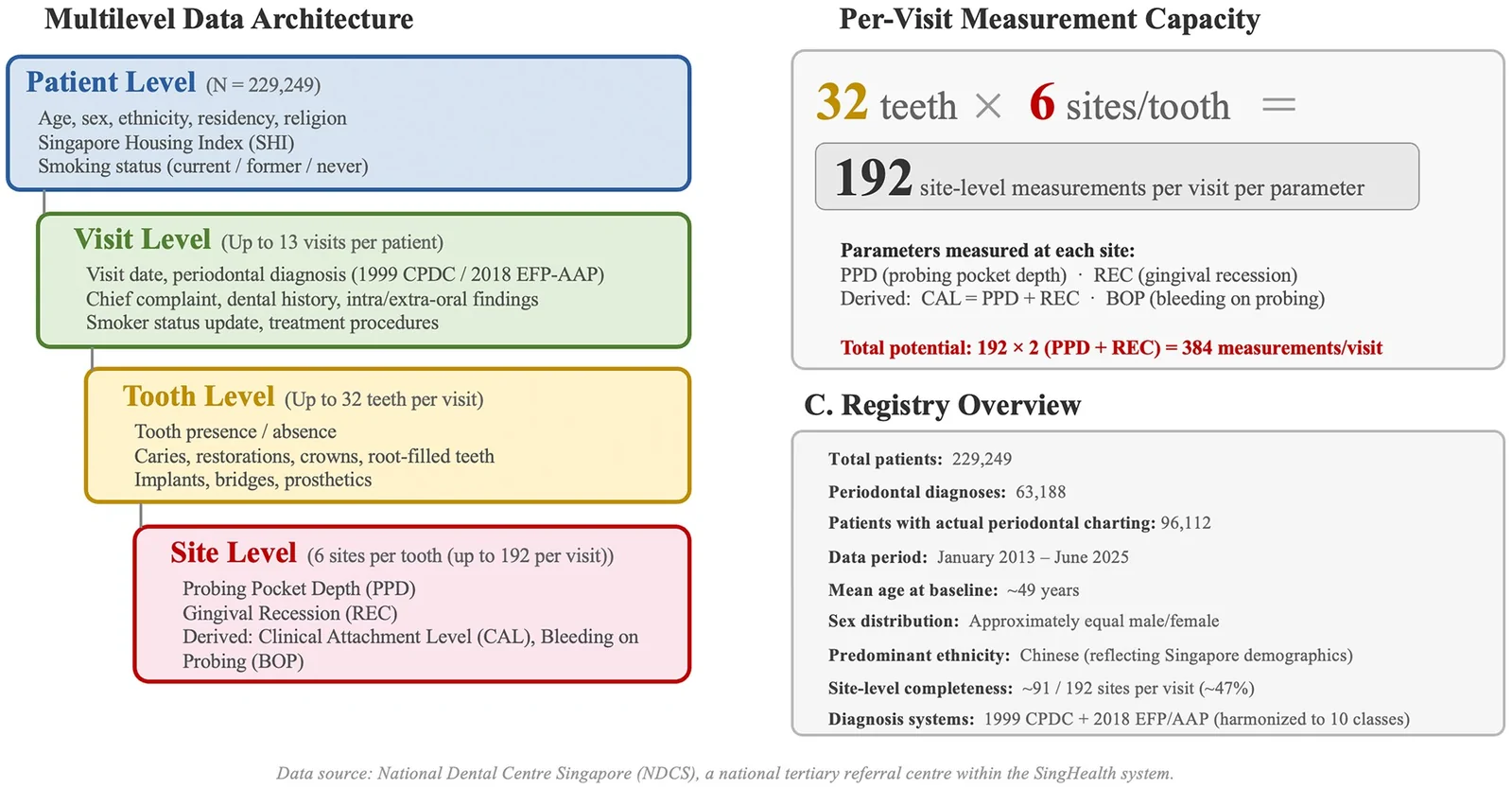
Hierarchical data structure of the NDCS-ODR registry.

**Figure 2:**
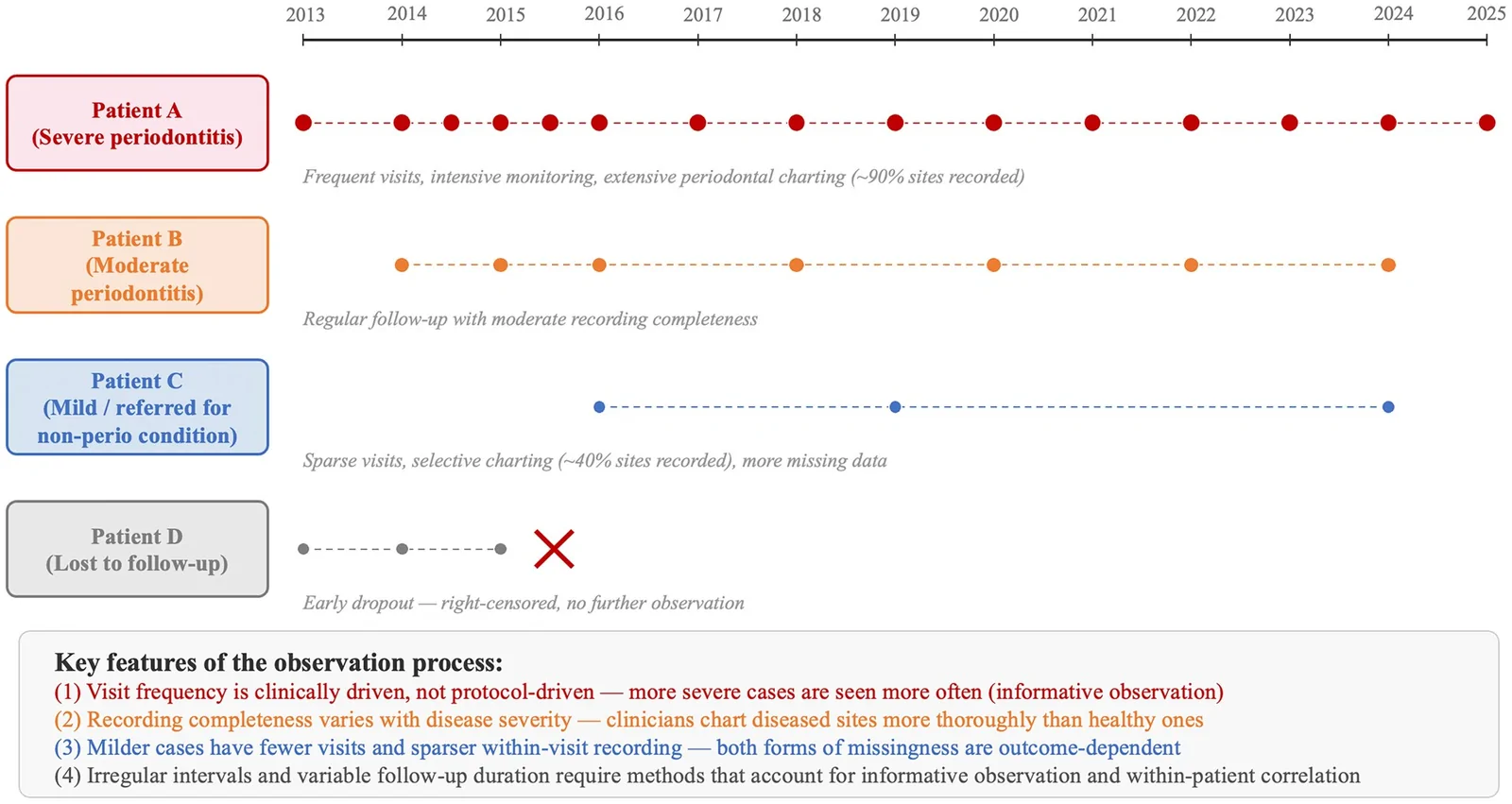
Real-world patterns of dental follow-up.

The registry captures a comprehensive set of structured clinical variables, organized within a hierarchical architecture comprising sites nested within teeth, teeth within visits, and visits within patients (Fig. 1). This multilevel structure reflects the biological organization of oral health and enables analyses that explicitly account for clustering within anatomical units and across time [15]. Figure 2 illustrates the hierarchical and longitudinal structure of the NDCS-ODR registry, showing how visit frequency and completeness of periodontal data vary according to patients’ periodontal status.

A distinctive feature of the registry is the availability of high-resolution periodontal data. Periodontal charting includes probing pocket depth (PPD), gingival recession (REC), and bleeding on probing (BOP), recorded at up to six sites per tooth across the full dentition, yielding 192 site-level observations per visit for each clinical parameter. The full structure of data collected is summarized in Table 2. These measurements allow precise characterization of disease severity, extent, and distribution, aligning with contemporary case definitions and staging frameworks for periodontitis [17, 18].

**Table 2:** Summary of data collected in the NDCS-ODR.

Domain	Level	Variables/measures	Description	Analytical relevance
Demographics and socioeconomic	Patient	Age, sex, ethnicity, residency status, religion, SHI	Individual characteristics captured at baseline and updated across visits; SHI used as proxy for socioeconomic position	Enables assessment of social gradients, inequalities, and life-course effects
Behavioural risk factors	Patient	Smoking status (current, former, never)	Longitudinally updated exposure variable	Key confounder and effect modifier in periodontal and systemic health analyses
Clinical diagnoses	Visit	Periodontal diagnoses (1999 CPDC; 2018 EFP/AAP), other oral conditions	Diagnoses harmonized across classification systems over time	Allows temporal comparisons and disease stratification
Clinical examination	Visit	Chief complaint, dental history, intra- and extra-oral findings	Structured clinical assessment data	Supports phenotyping and future integration with AI/NLP methods
Odontogram (tooth status)	Tooth	Tooth presence/absence, caries, restorations, crowns, root-filled teeth, prosthetics, implants	Comprehensive tooth-level status recorded at each visit	Enables analysis of tooth survival, treatment burden, cumulative disease experience
Periodontal charting	Site (nested within tooth)	PPD, REC, CAL, BOP, measured at six sites per tooth (up to 192 sites/visit)	High-resolution clinical measurements capturing current and cumulative periodontal status	Core data for disease definition, severity classification, and trajectory modelling
Derived disease endpoints	Site/patient	Binary thresholds: PPD ≥5, ≥6 mm; CAL ≥3, ≥4, ≥5 mm	Standard epidemiological definitions derived from continuous measures	Enables prevalence estimation and comparability across studies
Disease burden metrics	Patient	Number and % of diseased sites, tooth loss indicators	Aggregated measures across the dentition	Captures overall disease burden and clinical relevance
Longitudinal measures	Patient/visit	Visit-to-visit change in disease counts, trajectories of PPD/CAL	Repeated observations across visits	Supports trajectory modelling and causal inference
Data structure	Multilevel	Sites (≤192) nested within teeth, within visits, within patients	Hierarchical and longitudinal data architecture	Requires multilevel and correlated-data methods

1999 CPDC: 1999 classification of periodontal diseases and conditions; 2018 EFP/AAP: 2018 European Federation of Periodontology/American Academy of Periodontology classification; BOP: bleeding on probing; CAL: clinical attachment level; PPD: probing pocket depth; REC: gingival recession; SHI: Singapore Housing Index; SD: standard deviation.

Contextual variables include demographics, the Singapore Housing Index as a validated proxy of household-level socioeconomic position [19], and behavioural risk factors such as smoking. The integration of such variables supports the investigation of social gradients and common upstream determinants, consistent with the broader NCD framework and the common risk factor approach [20, 21]. NDCS-ODR directly captures comprehensive NCD outcomes. NDCS-ODR contains a range of demographic, socioeconomic, behavioural (e.g. smoking), and clinical oral health variables that are recognized as common determinants of both oral diseases and major NCDs. Through linkage with other SingHealth EHRs and national administrative databases, the registry can also be enriched with information on medical diagnoses, hospitalizations, medications, laboratory investigations, and mortality. This enables future studies to investigate shared risk factors, multimorbidity, and the relationships between oral health and chronic conditions such as diabetes, cardiovascular disease, chronic kidney disease, and other NCDs.

Medical history recorded during dental visits includes physician-diagnosed systemic conditions, medications, allergies, and medical devices relevant to dental care. These data can be supplemented through linkage with the SingHealth enterprise EHR, enabling comprehensive ascertainment of comorbidities and longitudinal medical outcomes.

### Data structure and completeness

The NDCS-ODR has substantially and systematically patterned missing data, reflective of real-world records. On average, approximately 91 out of 192 possible site-level measurements are recorded per visit, corresponding to roughly 53% missing data. This incompleteness arises from a combination of factors, including time constraints, clinical priorities, and patient-related considerations, reflecting the realities of clinical workflows in real-world settings. This missingness may not be random. Clinical recording tends to prioritize sites suspected to be diseased, generating outcome-dependent missingness that can lead to biased prevalence and trajectory estimates if not appropriately addressed [16, 22].

**Figure 3:**
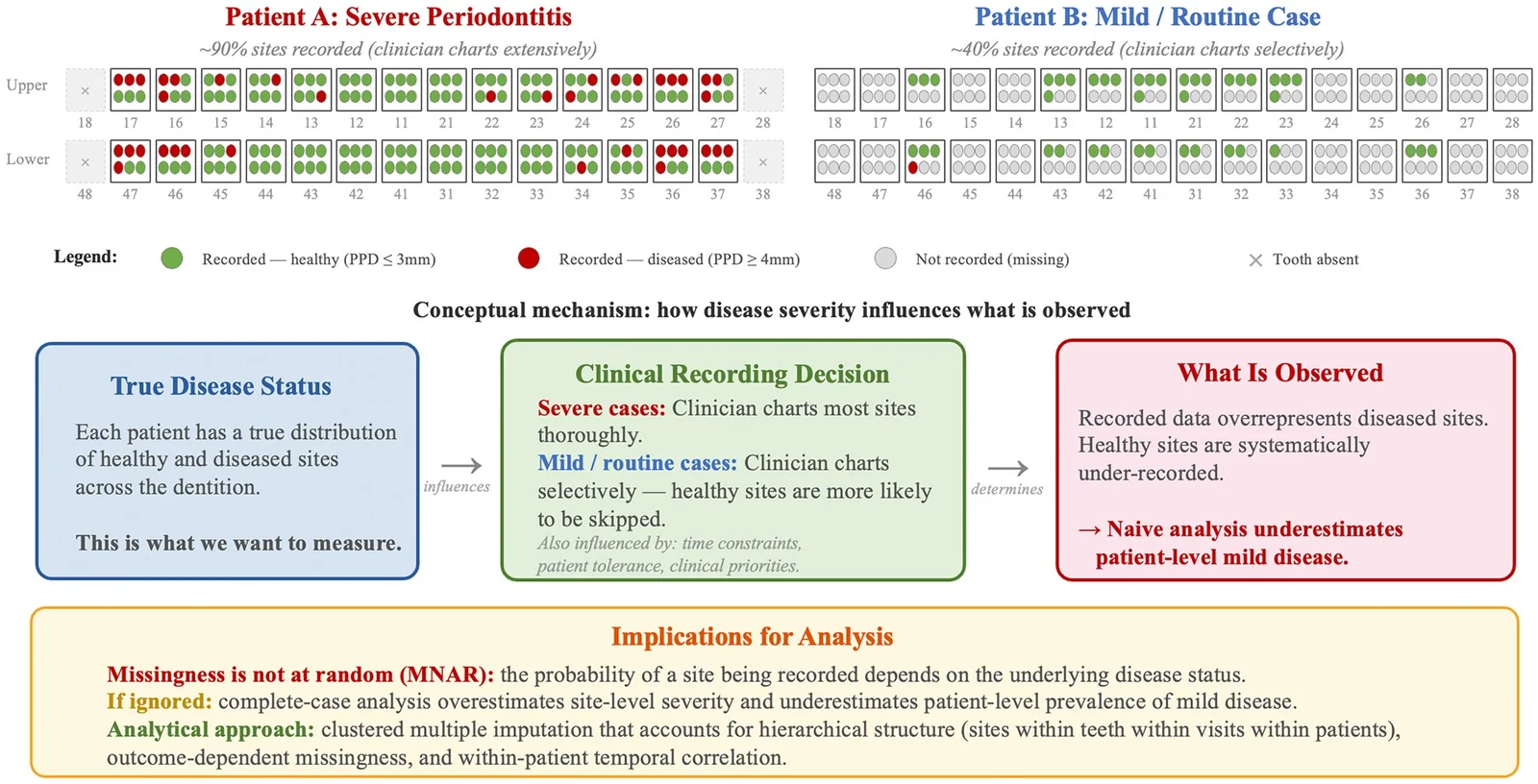
Preferential recording in real-world data.

Empirical analyses within the NDCS-ODR illustrate these implications. When site-level clinical attachment level [CAL] ≥ 3 mm was derived using only recorded observations, patient-level disease counts were substantially underestimated compared with estimates accounting for missingness, with the largest discrepancies observed for mild-to-moderate disease categories (Fig. 3). This pattern reflects the preferential recording of more severe sites in routine care and highlights the importance of explicitly modelling the observation process [2].

### Linkage

The NDCS-ODR sits within the SingHealth data ecosystem, enabling linkage with other clinical datasets through institutional data governance processes. Linkage relies on the National Registration Identity Card (NRIC) number, Singapore’s unique national identifier, which is recorded for every patient and matched deterministically across visits and against external datasets. To protect confidentiality, matching is done on the NRIC first; the identifier is then replaced by an encrypted pseudonym, so analysts receive only pseudonymized records. Re-identification is possible solely through a linkage file held by a separate custodian within SingHealth and requires specific approval, meaning that records can be linked without ever handling a directly identifying number. Because the NRIC is the common key across the SingHealth data ecosystem, any NRIC-keyed source can be linked in principle; to date, the registry has been linked to the SingHealth Diabetes Registry. This creates opportunities for research on oral–systemic relationships, multimorbidity, and healthcare utilization, extending the registry’s scope beyond oral health alone (Table 3).

**Table 3:** Linkable datasets.

Dataset	Examples of data available	Oral-health research rationale
SingHealth Diabetes Registry (SDR)	Diabetes status, HbA1c, glucose, glucose-lowering medications	Bidirectional periodontitis-glycaemia relationship; glycaemic control and tooth loss
SingHealth COPD and Asthma Data Mart (SCDM)	Asthma/COPD diagnosis, lung function, control/severity scores, inhaled and other medications	Possible effects of inhaled steroids on caries; periodontitis-respiratory associations
Perioperative and Anaesthesia Subject Area Registry (PASAR)	Pre-, intra-, and post-operative records, ASA physical status, ICU stay, 30-day and 1-year mortality	Pre-operative dental clearance
Laboratory results	HbA1c, CRP, and other inflammatory markers, lipids, renal function	Systemic-inflammation and metabolic correlates of periodontitis
Pharmacy/dispensing records	Antiresorptives (bisphosphonates, denosumab), immunosuppressants, xerostomia-inducing drugs	Medication-related osteonecrosis of the jaw; drug-induced dry mouth and caries risk
Hospital and polyclinic diagnoses (ICD/SNOMED)	Cardiovascular, renal, rheumatological and other chronic conditions	Multimorbidity and shared-risk-factor analyses
Admissions and emergency department attendances	Acute-care episodes, dental-related ED visits, length of stay	Healthcare utilization, avoidable dental presentations

ASA: American Society of Anesthesiologists; COPD: chronic obstructive pulmonary disease; CRP: C-reactive protein; ED: emergency department; HbA1c: glycated haemoglobin; ICD: International Classification of Diseases; ICU: intensive care unit; SNOMED CT: Systematized Nomenclature of Medicine—Clinical Terms.

## Data resource use

The registry supports applied epidemiology and health services research, including estimation of disease prevalence and severity, investigation of longitudinal trajectories and treatment outcomes, and examination of social gradients in oral health within a clinical population. Integration within SingHealth enables study of oral–systemic relationships and multimorbidity. As an illustrative example, ongoing work within the registry examines periodontal disease trajectories in patients with diabetes, linking repeated site-level CAL measurements to glycaemic control and treatment pathways under a target trial emulation framework. A second line of work evaluates the association between glycaemic control and tooth loss, leveraging repeated clinical measurements across visits to characterize how fluctuations in glycaemic control relate to cumulative tooth loss over follow-up.

Beyond these substantive applications, the NDCS-ODR serves as a methodological platform for addressing missing data in complex clinical datasets. Clustered multiple imputation frameworks have been developed using the registry to recover missing binary outcomes in high-dimensional longitudinal EHRs while preserving within-cluster correlation and temporal structure. Empirical analyses have demonstrated that reliance on observed data alone substantially underestimates disease burden, particularly for milder disease less likely to be recorded, whereas approaches that explicitly account for hierarchical dependence and informative missingness yield more accurate and internally consistent estimates.

The longitudinal depth and clinical granularity of the registry also support the application of target trial emulation principles to align observational analyses with causal questions, addressing time-varying confounding and informative observation processes in real-world data [2]. The NDCS-ODR is a relatively new data resource, and its detailed architecture, governance framework, data model, and implementation have been described in a dedicated methodological paper [23]. Ongoing projects include analyses of periodontal disease trajectories, evaluation of treatment pathways, and methodological work on outcome-dependent missingness in hierarchical longitudinal data.

## Strengths and weaknesses

The registry captures key contextual variables, including socioeconomic position, measured by the Singapore Housing Index, and behavioural risk factors such as smoking. Integrating these variables is a critical strength. This allows investigation of social and behavioural determinants of oral health within a clinical population. The approach aligns with the NCD framework, which recognizes strong social gradients and shared risk factors.

The NDCS-ODR offers several important advances over existing oral health surveillance systems and clinical registries. Unlike most national initiatives—such as NHANES (USA) and the Adult Dental Health Survey (UK)—which rely on periodic cross-sectional surveys with partial recording protocols and limited clinical detail, the NDCS-ODR can capture disease trajectories, treatment pathways, and within-person variability over time [24, 25]. Thus, while current systems are vital for population monitoring, the NDCS-ODR addresses key limitations by providing more comprehensive and longitudinal insights.

Clinical registries in oral health are comparatively underdeveloped and often focus on specific conditions, procedures, or service-delivery metrics, with limited integration of longitudinal follow-up and contextual determinants. Even where electronic dental records are available, their use for research is frequently constrained by fragmentation, lack of standardization, and limited capture of high-resolution clinical data across multiple levels of biological organization.

In contrast, the NDCS-ODR integrates longitudinal EHR data. It includes detailed site-level clinical measurements and contextual variables within one data architecture. This enables the simultaneous examination of disease progression, treatment patterns, and social determinants of oral health in a real-world clinical population. The registry also captures both clinical and data-generating processes, including outcome-dependent recording. As a result, it extends beyond traditional surveillance models and supports methodological innovation.

As such, the NDCS-ODR bridges a critical gap between population-based oral health surveillance and routine clinical data systems, aligning with calls to strengthen oral health information systems and integrate oral health within the broader NCD agenda [26–29]. Its design supports not only descriptive epidemiology but also longitudinal, causal, and implementation-focused research, positioning it as a next-generation oral health registry.

The NDCS-ODR has several important strengths that position it as a uniquely valuable resource for both epidemiological and methodological research. Its scale—over 229 000 individuals—and longitudinal design provide substantial statistical power and enable the investigation of disease dynamics, trajectories, and treatment responses over time. The high-resolution nature of the data, with measurements recorded at up to 192 sites per visit, allows for an unprecedented level of detail in characterizing disease distribution and severity within individuals, surpassing what is typically achievable in large-scale observational studies.

A further major strength lies in the integration of the registry within the SingHealth data ecosystem, which facilitates linkage with other clinical datasets and supports research on multimorbidity, health services utilization, and oral-systemic relationships. This aligns with the growing emphasis on real-world data as a cornerstone of contemporary epidemiology and health systems research [12–14]. Importantly, the NDCS-ODR goes beyond serving as a passive data repository. Its explicit recognition and systematic investigation of missing data processes—particularly outcome-dependent and hierarchical missingness—represent a distinctive contribution to methodological epidemiology. By providing a real-world setting in which data-generating mechanisms can be studied and modelled, the registry enables the development and validation of advanced analytical approaches for complex longitudinal data.

Several limitations must be considered. As a tertiary care dataset, the NDCS-ODR is not representative of the general population, and findings require cautious interpretation when extrapolated to broader populations. Follow-up is determined by routine clinical care rather than a fixed protocol, resulting in irregular visit intervals and informative observation processes that can introduce selection bias if not addressed [15]. Substantial outcome-dependent missingness is both a limitation and a defining feature; naïve analytical approaches may yield biased estimates, though this also creates opportunities for methodological innovation [22, 30]. Finally, certain data domains—including radiographs and unstructured clinical notes—are not currently captured, though these represent planned areas for future registry expansion.

## Data resource access

The NDCS-ODR operates within a secure governance framework compliant with Singapore’s regulatory requirements for data protection and biomedical research. Data are de-identified and stored within secure institutional computing environments, with access restricted to authorized users.

Researchers interested in using the registry submit a proposal outlining their intended analysis. Proposals are reviewed by the ODR Executive Committee, and access is granted following approval. External collaborators may participate through partnerships with ODR investigators. All analyses are conducted within secure institutional systems. Enquiries regarding collaboration and access can be directed to the National Dental Research Institute Singapore: ndris@ndcs.com.sg or to John Rong Hao Tay (john.tay.r.h@singhealth.com.sg).

## Ethics approval

The NDCS-ODR was approved by the SingHealth Centralised Institutional Review Board (CIRB Reference Code: 2022/2167) with a waiver of informed consent.

## Authors contributions

M.A.P. conceived the registry and drafted the manuscript. P.L. contributed methodological input on clustered imputation and missing data analyses. J.R.H.T. oversees the registry and contributed to interpretation, drafting, and critical revision. All authors approved the final version.

## Supplementary Material

dyag171_Supplementary_Data

## Data Availability

See ‘Data resources access’ above.

## References

[1] Hernán MA , Robins JM. Using big data to emulate a target trial when a randomized trial is not available. Am J Epidemiol 2016;183:758–64. 10.1093/aje/kwv254 PMC4832051 26994063

[2] Hernán MA , Robins JM. Causal Inference. Boca Raton, FL: CRC, 2010.

[3] Sim CPC , Lee YH, Sim YF et al Findings from the 2019 nationally representative oral health survey for adults in Singapore. Community Dent Oral Epidemiol 2024;52:281–91. 10.1111/cdoe.12961 38747365

[4] Kassebaum NJ , Smith AGC, Bernabé E et al; GBD 2015 Oral Health Collaborators. Global, regional, and national prevalence, incidence, and disability-adjusted life years for oral conditions for 195 countries, 1990-2015: a systematic analysis for the global burden of diseases, injuries, and risk factors. J Dent Res 2017;96:380–7. 10.1177/0022034517693566 PMC5912207 28792274

[5] Bernabe E , Marcenes W, Hernandez CR et al; GBD 2017 Oral Disorders Collaborators. Global, regional, and national levels and trends in burden of oral conditions from 1990 to 2017: a systematic analysis for the global burden of disease 2017 study. J Dent Res 2020;99:362–73. 10.1177/0022034520908533 PMC7088322 32122215

[6] Tay JRH , Li H, Leite FRM et al Tooth loss and masticatory function in older adults: a 15-year population-based time trend study in Singapore. J Dent 2026;165:106297. 10.1016/j.jdent.2025.106297 41391754

[7] Li H , Tay JRH, Malhotra R, Ma S, Peres MA. Subjective masticatory function and healthy life expectancy among older adults in Singapore: a multistate life table analysis. J Dent 2026;169:106655. 10.1016/j.jdent.2026.106655 41887495

[8] Tay JRH , Cooray U, Chan A, Tonetti MS, Nascimento GG, Peres MA. Perceived masticatory function and mortality: a causal study. J Dent Res 2026;105:95–102. 10.1177/00220345251363851 PMC12701900 40947960

[9] Tay JRH , Nascimento GG, Chan A, Malhotra R, Tonetti MS, Peres MA. Estimating the causal impact of chewing disability on depressive symptoms mediated by loneliness: a longitudinal marginal structural model study of older adults in Singapore. Innov Aging 2025;9:igaf100. 10.1093/geroni/igaf100 PMC12558686 41158119

[10] VanderWeele T. Explanation in Causal Inference: Methods for Mediation and Interaction. New York, NY, USA: Oxford University Press, 2015.

[11] Chow DY , Tay JRH, Nascimento GG. Systematic review of prognosis models in predicting tooth loss in periodontitis. J Dent Res 2024;103:596–604. 10.1177/00220345241237448 38726948

[12] Wanyonyi KL , Radford DR, Gallagher JE. Electronic primary dental care records in research: a case study of validation and quality assurance strategies. Int J Med Inform 2019;127:88–94. 10.1016/j.ijmedinf.2019.04.007 31128836

[13] Song M , Liu K, Abromitis R, Schleyer TL. Reusing electronic patient data for dental clinical research: a review of current status. J Dent 2013;41:1148–63. 10.1016/j.jdent.2013.04.006 PMC4141471 23603087

[14] Gordon SM , Camargo GA, Mejia GC, Sutherland JN. Use of the dental electronic health record for research: assessing demographic and oral health characteristics data for clinic patients. J Dent Educ 2018;82:1249–57. 10.21815/JDE.018.130 30504461

[15] Diggle P. Analysis of Longitudinal Data. Oxford, UK: Oxford University Press, 2002.

[16] Sterne JA , White IR, Carlin JB et al Multiple imputation for missing data in epidemiological and clinical research: potential and pitfalls. BMJ 2009;338:b2393. 10.1136/bmj.b2393 PMC2714692 19564179

[17] Tonetti MS , Greenwell H, Kornman KS. Staging and grading of periodontitis: framework and proposal of a new classification and case definition. J Periodontol 2018;89:S159–72. 10.1002/JPER.18-0006 29926952

[18] Papapanou PN , Sanz M, Buduneli N et al Periodontitis: consensus report of workgroup 2 of the 2017 world workshop on the classification of periodontal and peri-implant diseases and conditions. J Periodontol 2018;89:S173–82. 10.1002/JPER.17-0721 29926951

[19] Lim DYZ , Wong TH, Feng M, Ong MEH, Ho AFW. Leveraging open data to reconstruct the Singapore Housing Index and other building-level markers of socioeconomic status for health services research. Int J Equity Health 2021;20:218. 10.1186/s12939-021-01554-8 PMC8489093 34602083

[20] Marmot M. Social determinants of health inequalities. Lancet 2005;365:1099–104. 10.1016/S0140-6736(05)71146-6 15781105

[21] Sheiham A , Watt RG. The common risk factor approach: a rational basis for promoting oral health. Community Dent Oral Epidemiol 2000;28:399–406. 10.1034/j.1600-0528.2000.028006399.x 11106011

[22] Little RJ , Rubin DB. Statistical Analysis with Missing Data. Hoboken, NJ, USA: John Wiley & Sons, 2019.

[23] Tay JRH , Nascimento GG, Ho JSH et al Establishment of a large-scale oral disease registry (NDCS-ODR) in a national specialty center. PLoS One 2026;21:e0341766. 10.1371/journal.pone.0341766 PMC13345257 42418476

[24] Eke PI , Dye BA, Wei L et al Update on prevalence of periodontitis in adults in the United States: NHANES 2009 to 2012. J Periodontol 2015;86:611–22. 10.1902/jop.2015.140520 PMC4460825 25688694

[25] Steele J , O’Sullivan I. Executive Summary: Adult Dental Health Survey 2009. London: The Health and Social Care Information Centre, 2011, 5–22.

[26] Watt RG , Daly B, Allison P et al Ending the neglect of global oral health: time for radical action. Lancet 2019;394:261–72. 10.1016/S0140-6736(19)31133-X 31327370

[27] Petersen PE , Ogawa H. The global burden of periodontal disease: towards integration with chronic disease prevention and control. Periodontol 2000 2012;60:15–39. 10.1111/j.1600-0757.2011.00425.x 22909104

[28] Tay JRH , Bosworth HB, Fox CH et al Implementation research is underdeveloped in oral health: bridging the gap. J Dent Res 2026;105:570–7. 10.1177/00220345251410462 PMC13068148 41711035

[29] Peres MA , Jin L, Nascimento GG et al Periodontal diseases as a public health challenge: rationale, prevention, equity, and sustainable care-a global call for action. J Periodontal Res 2026. 10.1111/jre.70133 42313429

[30] Seaman SR , White IR. Review of inverse probability weighting for dealing with missing data. Stat Methods Med Res 2013;22:278–95. 10.1177/0962280210395740 21220355

